# A Cross‐Sectional Look at HPV Vaccine Acceptance and Associated Factors Among Male University Students in Lebanon

**DOI:** 10.1155/aiu/9303212

**Published:** 2025-12-19

**Authors:** Ziad Koberssy, Joey El Khoury, Rami Halabi, Joviane Daher, Nadine Saleh, Raghid El Khoury, Charbel El Hachem

**Affiliations:** ^1^ Notre Dame des Secours University Hospital Center, Street 93, Byblos, Postal Code 3, Lebanon; ^2^ Department of Urology, School of Medicine and Medical Sciences, Holy Spirit University of Kaslik, P.O. Box 446, Jounieh, Lebanon, usek.edu.lb; ^3^ Faculty of Public Health, Lebanese University, Fanar, Lebanon, ul.edu.lb

**Keywords:** HPV vaccination, knowledge barriers, male university students, sexually transmitted infections

## Abstract

**Background and aims:**

The human papillomavirus (HPV) is the leading sexually transmitted infection (STI) worldwide. Oncogenic HPV serotypes lead to various anogenital cancers in men. Societal changes in previously conservative regions like Lebanon have increased STI risks, particularly among university students. While several studies have focused on HPV prevalence in Lebanese females, data on males remain scarce. Men HPV vaccination is crucial for the prevention of HPV‐related cancers in both sexes. This study aims to assess vaccination prevalence among Lebanese male university students, identify influencing factors, and evaluate their HPV and its vaccine knowledge.

**Methods:**

This is a cross‐sectional analytical study surveying male students at Lebanese universities ranging from 17 to 30 years. Data were collected using closed‐ended questions distributed through an online platform. We compared vaccination rates with different variables using a bivariate analysis. The chi‐squared test was utilized for categorical variables, while Fisher’s exact test was employed for nonparametric evaluations of categorical data. For continuous variables, parametric and nonparametric analyses, the independent sample *t*‐test, and the Mann–Whitney *U* test, respectively, are used. Throughout these investigations, statistical significance was set at a *p*‐value ≤ 0.05 (95% confidence interval).

**Results:**

324 individuals completed the study. HPV vaccination prevalence was 5.2% (*n* = 17). In people not taking the HPV vaccine, limited knowledge of HPV (34.57%) and its vaccine (25.62%) as well as the lack of physician recommendation (31.17%) were the main barriers. Compared to their nonvaccinated peers (3.27 over 13), vaccinated participants (6 over 13) showed an increased HPV vaccination knowledge index (*p* = 0.03).

**Conclusion:**

The study revealed diminished HPV vaccination rates among male university students in Lebanon, primarily attributed to inadequate knowledge and low physician recommendations. Targeted awareness campaigns and incorporating HPV vaccination into the national vaccination card are recommended to promote vaccination rates.

## 1. Introduction

The human papillomavirus (HPV) is a member of the Papillomaviridae family, a double‐stranded DNA virus, classified among the eight most incident STIs according to the WHO [1]. Humans are the only reservoir of HPV, and possible modes of transmission include all types of sexual activities, even close skin‐to‐skin touching during sex, with the highest incidence through vaginal or anal sex [2, 3]. More than 200 discernible serotypes of HPV are classified as oncogenic (predominantly HPV 16 and HPV 18) and are causative of anogenital cancers, or nononcogenic serotypes (HPV 6 and HPV 11) associated with warts [3]. According to the National Cancer Institute (NIH), HPV is linked to at least six types of cancer. It is the causative agent in nearly all cervical cancers, more than 90% of anal cancers, and approximately 70% of oropharyngeal cancers. Furthermore, HPV is identified as the causative agent in 75%, 69%, and 63% of vaginal, vulvar, and penile cancers, respectively [3].

Notably, HPV ranked first among STIs worldwide [4], with an estimated occurrence of at least once in lifetime infection among sexually active men and women [5]. A systematic review found that, depending on populations, men HPV global prevalence ranges from 1.3% to as high as 72.9% with most of the studies showing a prevalence of 20% or more [4]. Research has primarily focused on HPV infection among women, rather than men, potentially due to its association with cervical cancer; however, prevalence of HPV among men was as high as in women [4]. Moreover, men’s HPV infection can contribute to the transmission of the virus to their female partners, potentially increasing the risk of women’s cervical cancer. Therefore, addressing HPV in men not only helps reduce overall transmission but also contributes to reducing cervical cancer incidence in women and facilitates management of affected female patients [6]. In previously known conservative societies, including Lebanon, where HPV prevalence was relatively low, globalization‐induced lifestyle changes increased the risk of STIs; currently, the younger demographic is increasingly liberal and less rigidly adherent to its sociocultural conventions compared to earlier years [7]. Hence, Lebanese university students are showing risky sexual behaviors coupled with reduced contraceptive use and limited awareness of STI prevention measures [8, 9]. Large studies assessing the prevalence of HPV infection in Lebanon are missing, especially among males or university students. In a study done on 1026 Lebanese females, the authors found that HPV infection prevalence is up to 5% among this group [10, 11]. According to a recent report, the crude incidence rate of cervical cancer in Lebanon is 3.66 per 100,000 women per year, with approximately 124 new cases and 73 deaths annually. Cervical cancer is ranked as the 8th most frequent cancer among young women in Lebanon. In males, anal cancer, penile cancer, and oropharyngeal incidence rates were 0.17, 0.26, and 0.12, respectively [11]. Additionally, Lebanon had the highest rate of HPV positivity among oropharyngeal squamous cell carcinoma (OPSCC) cases in a 2021 systematic review among 26 countries, with 85% of OPSCC tested positive for HPV in Lebanon [12]. These numbers may under‐represent the real HPV prevalence, since incomplete cancer registration, lack of screening programs for male HPV‐related cancers, and variable diagnostic capacity may lead to underestimation of true incidence, particularly in low‐ and middle‐income countries [13, 14].Multiple studies have highlighted the increased risk of HPV infection among men who have sex with men (MSM) [15]. People with an increased number of sex partners had an increased risk of HPV infection as well, while condom use and circumcision were identified as potential protective factors [16, 17]. HPV vaccination remains the cornerstone in HPV prevention, providing up to 90% prevention of HPV‐related cancers [3]. The newly FDA approved, and the only vaccine used in the USA since 2016, Gardasil 9, offers immunity against nine strains (6‐11‐16‐18‐31‐33‐45‐52‐58), accounting for up to 90% of cervical cancers and other HPV‐caused cancers [18]. The quadrivalent HPV vaccine significantly reduced the incidence of external genital lesions in young males, including precancerous penile and anal lesions, associated with HPV types 6, 11, 16, and 18 [19]. Additionally, this vaccine was associated with a significant reduced risk of cervical cancer in young women, while not yet shown directly in men; similar reductions are anticipated for male HPV‐related cancers [20]. In 2019, the Advisory Committee on Immunization Practices of the Centers for Disease Control and Prevention (CDC’s ACIP) recommended routine vaccination against HPV at ages 11–12 years or for everyone through 26 years if not immunized adequately at younger age [21]. Although approved by the FDA, the vaccine is not recommend by the ACIP for individuals aged 27–45 years, as benefits are limited in this age group; instead, a communication with the physician is encouraged [21]. For adults aged > 45 years, the HPV vaccine is unlicensed [21]. The CDC’s ACIP also recommends receiving 2 doses if vaccination starts before the age of 15 and receiving the 3‐dose schedule in case vaccination starts at ages 15 through 45 years [21]. These recommendations highlight the importance of HPV vaccination at younger ages due to a stronger immune response and vaccination prior to exposure to the virus, which emphasizes the importance of increasing awareness among university students regarding HPV vaccination.

Despite its efficacy, vaccination rates remain low among Lebanese population; 16.7%–18.9% of Lebanese female university students were vaccinated according to two studies published in the literature [7, 22]. As for Lebanese secondary school students, in 2023, the vaccination rate was 15.2% and 4.6% among females and males, respectively [23]. These low vaccination rates in Lebanon may be attributed to the possibly expensive cost of HPV vaccination, alongside other possible factors [7, 22]. To our knowledge, studies assessing vaccination attitudes among Lebanese male university students are lacking, and insufficient international data are available about this topic. Consequently, our study’s objective is to determine the prevalence of vaccine administration in male Lebanese university students nationwide and to discover predictors that may influence the decision of vaccine uptake. Additionally, we aim to compute novel HPV and HPV vaccination knowledge scores. Reaching our objectives is key for a potential awareness campaign on a national scale and conceivably adding the HPV vaccine to the Lebanese National vaccination card.

## 2. Methods

### 2.1. Study Design

We carried out an analytical cross‐sectional study from December 2022 through February 2024. Data were collected through an online self‐administered questionnaire using Microsoft Forms.

### 2.2. Ethical Consideration

Our study received approval from the Institutional Review Board of Notre Dame des Secours‐University Hospital (NDS‐UH IRB) on December 13, 2022. We conducted a completely anonymous data collection ensuring the absence of any personal or confidential details. Responders were presented at the beginning of the survey with an introductory text outlining the study’s subject and the objectives. Subsequently, they were provided the option to proceed or decline participation in the form of informed consent.

### 2.3. Study Population and Sampling

The inclusion criteria established for our study were males who are university students, aged 17–30 years, and living in Lebanon. We employed the “snowball” sampling technique with a “cascading progression.” We sent messages containing a URL to our questionnaire, accompanied by an informative text outlining our study’s objectives, to class representatives and students from all governorates to assist in our sampling efforts and spread our survey to their peers.

### 2.4. Minimal Sample Size Calculation

We employed Epi Info software v7.2 for our calculations. Given that Lebanese universities enrolled a total of 107,268 male students [24], and considering that the anticipated prevalence of HPV vaccination among MENA men (59% had Lebanese Heritage) was 23.2% [25], our calculated minimum sample size, with a confidence level of 95% and an acceptable margin of error of 5%, was determined to be 273.

### 2.5. Questionnaire

The questionnaire was developed after conducting a thorough review of the existing literature pertinent to our research objectives. Before distributing the definitive version, we sought specialized perspectives to ensure the questionnaire’s effectiveness, relevance, and clarity. Preliminary testing was conducted prior to surveying to ensure clear question phrasing and assess the average completion time, which was found to be 10 min. Comprising a total of 78 questions, the questionnaire was structured into eight distinct sections. The first four segments were dedicated to gathering information on participants’ sociodemographic aspects and behavioral characteristics, medical history, and sexual behavior. Following these initial sections, subsequent parts of the questionnaire delved into responders’ attitudes toward HPV vaccination. Additionally, we added questions to assess participants’ knowledge regarding HPV and its vaccine.

### 2.6. Variables and Definitions

#### 2.6.1. Primary Outcome

The primary outcome of the study was male HPV vaccination prevalence. This was defined as the proportion of male participants who reported having received at least one dose of the HPV vaccine at the time of data collection. This variable served as the main outcome measure for evaluating HPV vaccine uptake in the study population.

#### 2.6.2. Secondary Outcome

To assess knowledge levels regarding HPV and the HPV vaccine, two indices were computed. Each correct single‐response question garnered 1 point toward the respective index. The maximum HPV knowledge score was 23, while the maximum score of HPV vaccine knowledge was 13.

Independent variables are demographic characteristics, sexual behavior, and parental education.

#### 2.6.3. Prespecified Analyses

Associations between vaccination uptake and sociodemographic factors.

Statistical analyses were performed using SPSS v.29 (IBM SPSS Inc., Chicago, IL, USA), the acquired data underwent processing, including compilation, coding, and subsequent analysis of variables. We used mean values accompanied by their respective standard deviations (SDs) for the presentation of continuous variables. On the other hand, frequencies and percentages were employed to summarize categorical variables. Bivariate analyses were executed; chi‐squared test was utilized for categorical variables, while Fisher’s exact test was employed for nonparametric evaluations. Furthermore, concerning continuous variables, for parametric and nonparametric analyses, the independent sample *t*‐test and the Mann–Whitney *U* test were used, respectively. For all analyses, a *p*‐value threshold of ≤ 0.05, with a 95% confidence interval, was determined statistically significant.

## 3. Results

### 3.1. Descriptive

The sociodemographic characteristics, sexual behaviors, and selected health‐related features of the study participants.

324 participants met the inclusion criteria and successfully completed the questionnaire. Age ranged from 17 to 30 years, with 22.1 years (±3.1) being the average mean of age. Most participants are pursuing majors in nonmedical fields (70.4%). Participants were predominantly enrolled in private Lebanese Universities (87.3%) compared to the public Lebanese Universities (12.7%). Nearly half of the participants had a maternal (54.6%) and paternal (46.9%) educational level of a university degree or higher. Regarding medical background, 2.5% only reported cancer(s) related to HPV in their familial history. Regarding behavioral characteristics, 37.7% of the students frequently consumed alcohol, while almost two‐thirds of them (63.3%) did not smoke. Concerning sexual practices, approximately two‐thirds of the participants (67.6%) reported not being sexually active, and most participants (93.8%) never had a prior history of STI. In counterpart, among currently sexually active participants, only 6.5% were engaged exclusively in sexual activities with other men. Almost half of the students selected vaginal sex as their preferred sexual practice. Detailed findings regarding sociodemographic, sexual, and behavioral aspects of responders are summarized in Table 1.

**Table 1:** The sociodemographic characteristics, sexual behaviors, and selected health‐related features of the study participants.

	Mean ± SD or *n* (%)	
*Sociodemographic characteristics*		
Age (years)		22.1 ± 3.1
Major	Medical^1^	96 (29.6%)
	Nonmedical	228 (70.4%)

University	Public	41 (12.7%)
	Private	283 (87.3%)

Family Status	Single	252 (77.8%)
	In a Relationship/Married	72 (22.2%)

Residence	Beirut	33 (10.2%)
	Mount Lebanon	212 (65.4%)
	Other^2^	79 (24.4%)

Religion	Christian	275 (84.9%)
	Muslim/Druze	49 (15.1%)

Maternal educational level	Less than university degree	147 (45.4%)
	University degree or higher	177 (54.6%)

Paternal educational level	Less than university degree	172 (53.1%)
	University degree or higher	152 (46.9%)

*Medical history*		
Family history of cancer‐related to HPV^3^	Yes	8 (2.5%)
	No History	316 (97.5%)

History of sexually transmitted disease	At least once	20 (6.2%)
	Never	304 (93.8%)

*Behavioral characteristics*		
Alcohol consumption	No	77 (23.8%)
	Frequently	122 (37.7%)
	Occasionally	125 (38.6%)

Smoking	Yes	119 (36.7%)
	No	205 (63.3%)

*Sexual characteristics*		
Age on first sexual intercourse (years)		18.2 ± 2.7
Number of partners in general	One	72 (22.2%)
	Two or more	33 (10.2%)
	Not sexually active	219 (67.6%)

Partner(s) gender	Man	21 (6.5%)
	Woman	120 (37.0%)
	Both	11 (3.4%)
	No sexual partner	172 (53.1%)

Sexual preference (multiple choice question)	Anal Giving	47 (14.5%)
	Anal Receiving	23 (7.0%)
	Oral Giving	69 (21.3%)
	Oral Receiving	68 (21.0%)
	Vaginal Sex	150 (46.3%)
	Not Sexually active	172 (53.1%)

Abbreviation: SD = standard deviation.

^1^“Medical students” include students in medicine, nursing, and pharmacy.

^2^Aakar, North, Baalbeck‐Hermel, Beqaa, South, Nabatiyeh.

^3^Cervical cancer, penile cancer, anal cancer, oropharyngeal cancer.

### 3.2. HPV Vaccination

**Figure 1:**
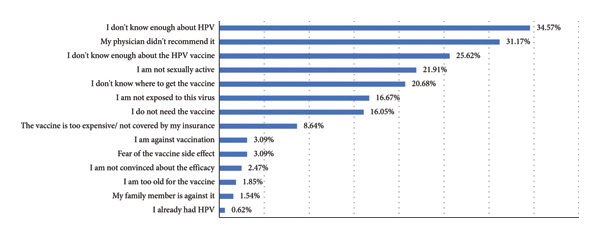
HPV vaccination restrictions.

We found that the prevalence of having at least one HPV vaccine dose was 5.2% with 4% proceeding to receive a second dose. The mean age at the time of receiving the initial dose was 17.8 years (±5.1 years) ranging from 11 years through 28 years of age. The primary reported factor for abstaining from taking the HPV vaccine was a lack of HPV awareness (34.57%), followed by the absence of a physician’s recommendation (31.17%), and unfamiliarity with the HPV vaccine (25.62%) (Figure 1). Table 2 provides an additional description of the variables associated with HPV vaccination. Moreover, Figure 1 illustrates constraints contributing to the absence of vaccine uptake.

**Table 2:** HPV vaccination variable details.

		Mean ± SD or *n* (%)
Age of HPV vaccination uptake (first dose) (years)		17.8 ± 5.1
Age of HPV vaccination uptake (first dose) in a participant with a history of sexual activity (years)		20.6 ± 4.5
HPV vaccine uptake (at least the 1^st^ dose)	Yes	17 (5.2%)
	No	307 (94.8%)

HPV vaccine uptake (second dose)	Yes	13 (4.0%)
	No	4 (1.2%)
	Did not take the first dose	307 (94.8%)

HPV vaccine uptake (third dose)	Yes	9 (2.8%)
	No	4 (1.2%)
	Did not take any doses	311 (96.0%)

Do you intend to get the HPV vaccine in the next year?	Yes	109 (33.6%)
	No	198 (61.1%)
	Already took a dose	17 (5.2%)

Have you ever heard about HPV?	Yes	155 (47.8%)
	No	169 (52.2%)

Have you ever heard about HPV vaccine?	Yes	132 (40.7%)
	No	174 (53.7%)

Abbreviation: SD = standard deviation.

### 3.3. HPV and Its Vaccination Knowledge

The mean score for the HPV knowledge index was 9.5 out of 23 (SD: ±5.7). In comparison, the HPV vaccine knowledge index mean score stood at 3.4 out of 13 (SD: ±3.7). Additionally, the knowledge index ranged from 3 to 22 for HPV and 0 to 12 for HPV vaccination, as detailed in Table 3.

**Table 3:** Evaluation of HPV and HPV vaccine awareness scores.

	Mean ± SD	Min	Max
HPV knowledge score^∗^	9.5 ± 5.7	0	22
HPV vaccine knowledge score^∗∗^	3.4 ± 3.7	0	12

^∗^The maximum score obtainable is 23.

^∗∗^The maximum score obtainable is 13.

### 3.4. Bivariate Analysis

Associations between HPV vaccine uptake and participant characteristics.

Table 4 presents statistical associations between HPV vaccine uptake and social‐demographic variables, medical background, and practices. Our findings indicate that medical students exhibit a higher likelihood (12.5%) of receiving a minimum of one dose of the HPV vaccine compared to their counterparts in nonmedical specialties (2.2%). Furthermore, a positive correlation was observed between higher maternal education and increased vaccination rates. Specifically, 8.5% of participants whose mothers had attained a university degree or higher were vaccinated, compared with 1.4% of those whose mothers had an education level below university. The study also revealed a negative correlation between alcohol consumption and vaccination rates. Notably, vaccination rates were 11.7%, 4.1%, and 2.4% for individuals with no, frequent, and occasional alcohol consumption, respectively.

**Table 4:** Associations between HPV vaccine uptake and participant characteristics.

		Vaccinated	Nonvaccinated	*p*‐value
		Mean ± SD or *n* (%)		
*Sociodemographic characteristics*				
Age		22.24	22.13	0.7 m
Major	Medical specialty^1^	12 (12.5%)	84 (87.5%)	< 0.01c
	Nonmedical specialty	5 (2.2%)	223 (97.8%)	

University	Public	0 (0%)	41 (100%)	0.14 f
	Private	17 (6%)	266 (94%)	

Family status	Single	14 (5.6%)	238 (94.4%)	0.7 f
	In a relationship/married	3 (4.2%)	69 (95.8%)	

Residence	Beirut	4 (12.1%)	29 (87.9%)	0.120 c
	Mount Lebanon	8 (3.8%)	204 (96.2%)	
	Other^2^	5 (6.3%)	74 (93.7%)	

Religion	Christian	12 (4.4%)	263 (95.6%)	0.153 f
	Muslim	5 (10.2%)	44 (89.8%)	

Maternal educational level	Less than university degree	2 (1.4%)	145 (98.6%)	0.004 c
	University degree or higher	15 (8.5%)	162 (91.5%)	

Paternal Educational Level	Less than university degree	6 (3.5%)	166 (96.5%)	0.131 c
	University degree or higher	11 (7.2%)	141 (92.8%)	

*Medical History*				
Cancer History	No	17 (5.3%)	303 (94.7%)	1.000 f
	Yes	0 (0%)	4 (100%)	

Family history of cancer related to HPV^3^	Yes	1 (12.5%)	7 (87.5%)	0.667 c
	No History	16 (5.1%)	300 (94.9%)	

History of STD	At least once	2 (10.0%)	18 (90.0%)	0.283 f
	Never	15 (4.9%)	289 (95.1%)	

*Habits*				
Alcohol drinking	No	9 (11.7%)	68 (88.3%)	0.012 f
	Frequently	5 (4.1%)	117 (95.9%)	
	Occasionally	3 (2.4%)	122 (97.6%)	

Smoking	Yes	6 (5%)	113 (95%)	0.9 c
	No	11 (5.4%)	194 (94.6%)	

Abbreviation: SD = standard deviation.

^1^ “Medical students” include students in medicine, nursing, and pharmacy.

^2^Aakar, North, Baalbeck‐Hermel, Beqaa, South, Nabatiyeh.

^3^Cervical cancer, penile cancer, anal cancer, oropharyngeal cancer.

### 3.5. HPV Vaccination and Sexual Behaviors

The detailed results appear in Table 5. Our study showed that men who have sex with men (MSM) had higher vaccination rates (19%) than heterosexual men (4.2%) or bisexual men (0%). Additionally, 19.2% of participants with two or more partners received the vaccine, in contrast to 3.2% of students engaged with only one partner.

**Table 5:** Bivariate analysis of sexual behavior questions.

		Vaccinated	Nonvaccinated	*p*‐value
		Mean ± SD or *n* (%)		
Age on the first sexual intercourse		17.6	18.2	0.547 t
Sexual activity	Active	9 (5.9)	143 (94.1)	0.609 c
	Not active	8 (4.7)	164 (95.3)	

Number of partners usually	One	4 (3.2)	122 (96.8)	0.003 c
	Two and more	5 (19.2)	21 (80.8)	
	Not sexually active	8 (4.7)	164 (95.3)	

Partner(s) gender	Man	4 (19)	17 (81)	0.029 c
	Woman	5 (4.2)	115 (95.8)	
	Both	0 (0)	11 (100)	
	Not sexually active	8 (4.7)	164 (95.3)	

*Note:* c: Chi‐squared test; t: Student′s *T*‐test; f: Fisher’s test.

### 3.6. Association Between Vaccine Uptake and Knowledge Scores

Table 6 delineates the correlation between vaccine uptake and knowledge scores. Vaccinated respondents demonstrated better knowledge about the HPV vaccine, with an average score of 6, as opposed to 3.27 out of 13 among their nonvaccinated counterparts.

**Table 6:** Bivariate analysis of vaccine uptake and knowledge scores.

	Vaccinated	Nonvaccinated	*p*‐value
	Mean		
HPV knowledge score	11.11	9.46	0.243 t
HPV vaccine knowledge score	6	3.27	0.003 t

*Note:* t: Student′s *T*‐test.

## 4. Discussion

To the best of our knowledge, no studies have been conducted to assess HPV vaccination among male Lebanese university students. Given that HPV vaccination appeared to be the only tool to reduce the increasing number of new HPV infection cases and to reduce subsequently HPV‐related cancers in men [26]. Our study also marks the first attempt to assess HPV and HPV vaccine knowledge among male Lebanese university students. Thus, we focused on filling this gap by examining HPV vaccine administration prevalence and its related determinants within a sample of 324 Lebanese male college students aged from 17 to 30 years. Furthermore, we intended to formulate valuable insights into factors influencing HPV vaccination administration by computing knowledge scores of HPV and HPV vaccines. Based on our findings, only 5.2% of our surveyed male students have undergone at least one HPV vaccination dose by the time of the survey, and approximately 2.8% have completed the entire recommended series of three consecutive doses. Notably, as mentioned above, the CDC advises a full regimen varying from 2 to 3 doses based on age, further, considering the absence of HPV vaccination schedule on the national vaccination calendar, we determined that the prevalence of HPV vaccination among the surveyed student population is 5.2%, acknowledging that a subset of this proportion may not have attained an optimal immunization status.

Comparable studies have been conducted and indicated a higher prevalence of vaccination among females compared to that of males. For instance, among female Lebanese university students, a study reported that 18.9% of their sample (*n* = 454) had received at least one dose of HPV vaccine [22], while a similar study conducted in 2015 found a vaccination rate of 16.5% among female college students at a Lebanese private university [7]. This discrepancy between the two genders is not unique to university settings but extends to secondary school students, where vaccination rates were 15.2% for females and 4.6% for males [23]. We assume that this gap between the two genders may be attributed to the fact that campaigns that promote HPV vaccination to prevent HPV‐related cancer primarily targeted cervical cancer protection, and consequently discussions about HPV and its vaccination centered around women [27]. Moreover, women usually make the decision when it comes to their children’s vaccination, reinforcing a perception among men that additional knowledge regarding HPV vaccination is unnecessary [27, 28]. Our findings align with other similar studies conducted in the Middle Eastern countries. In Turkey, two studies among 1723 male university students and 1160 students aged between 18 and 30 years reported that only 4.9% and 0.4%, respectively, took at least one dose of HPV vaccine [29, 30]. Another study assessing acceptability of HPV vaccine among 517 male Saudi Medical students found that only 8.7% had taken the vaccine [31]. Interestingly, a systematic review done in the United States of America (USA) found that HPV vaccination rates among US male university students, after FDA approval for male vaccination in 2009, varied between as low as 1.8% and as high as 56.3%, with most studies demonstrating vaccination rates less than 20% [32]. This systematic review highlights the fact that even in higher income and more liberal countries, such as the USA, male university students’ vaccination rates are still low and are significantly under the US 2020 Healthy People target, which was 80% [33].

In terms of knowledge scores concerning HPV, the index was low, with a mean score of 9.5 out of 23 (41.3%). Male knowledge about HPV is slightly lower than that of female Lebanese university students, whose mean score was 49.64% in previous studies [7, 22]. The same pattern is observed for the HPV vaccine knowledge score; male students scored an average of 3.4 out of 13 (26.2%), while studies on female counterparts reported an average score of 36.6% [17]. This lack of awareness was also evident among secondary school students [23], indicating that various genders and age groups in Lebanon lack sufficient knowledge about HPV and its vaccine.

Multiple reasons may be the culprit behind reduced vaccination rates among male college students. First, lack of awareness about HPV and its vaccine’s protective effect were the main barriers against vaccination. Indeed, 34.57% of respondents reported not receiving the vaccine because they were unfamiliar with HPV, and 25.62% reported a lack of knowledge about the vaccine itself. Additionally, in our study, we found that vaccinated students scored higher on HPV (11.11 vs. 9.46 out of 23) and HPV vaccination (6.0 vs. 3.27 out of 13) knowledge indices. It is worth noting that knowledge indices regarding HPV and its vaccine were low among both vaccinated and unvaccinated students. Plus, vaccination rates were higher among medical students (12.5%) compared to nonmedical students (2.2%), possibly due to the relatively higher level of knowledge. Similar trends are shown in the Turkish study where 49.5% of participants attributed their abstinence of vaccination to their lack of knowledge about the protective effect of the vaccine [30]. Moreover, Saudi male medical students thought that vaccination awareness initiatives would assist them in making decisions regarding acceptance of vaccine uptake [31]. Lack of knowledge was also considered a barrier against vaccination in multiple studies in the USA as depicted by the USA systematic review [32]. These findings emphasize the need to increase awareness about HPV vaccination among male students at Lebanese universities to improve vaccination rates; in fact, a systematic review of 12 studies conducted in the USA found that communication technologies, such as text messages, health record prompts, interactive videos, and emails targeting students, parents, or providers, effectively increased both vaccination initiation and completion [34]. We consider the critical role of healthcare providers and officials in implementing strategies to enhance awareness and promote HPV vaccination across diverse demographic segments in Lebanon.

Moreover, we found that another significant barrier to not opting for the vaccine is the absence of a physician’s recommendation (31.17%). This observation aligns with findings from other local and global studies, which consistently report that the lack of healthcare provider recommendation is one of the primary obstacles to HPV vaccination [35–37]. Physicians tend to recommend HPV vaccination primarily for adolescent females rather than males due to “feminization” of HPV emphasizing its role in protecting against cervical cancer [38, 39]. A study addressing physicians’ recommendations in Lebanon found a notable disparity, revealing that female patients were 6.8‐folds more susceptible to being advised for vaccination against HPV compared to males; the authors attributed this discrepancy to a possible limited comprehension among physicians in Lebanon concerning HPV‐related cancers in males beyond cervical cancer in females [38]. Therefore, improving physician education is crucial for increasing young men vaccination [37, 40]. Another possible factor contributing to the low recommendation rates might be physician’s uncertainty about the cost‐effectiveness ratio of the vaccine. In Lebanon, a study identified the expenses of the vaccine as a primary obstacle hindering physicians from recommending it [40]. Indeed, only 8.64% of students in our study did not take the vaccine because it is expensive; this could be best explained by the limited information about the vaccine’s cost. These findings raise the importance of incorporating HPV vaccination into the Lebanese national vaccination card. This approach ensures that all physicians can readily recommend it and facilitates coverage by third‐party payers. Interestingly, after introduction of HPV vaccination to the English immunization program in 2008, a substantial decrease in vaccine strains, including HPV 16/18, prevalence was seen [41]. By aligning HPV vaccination with CDC vaccination recommendations, we enhance its visibility and accessibility, promoting broader protection against HPV‐related diseases and cancer.

Another potential contributor to the low vaccination rates observed in our sample is the relatively low level of reported sexual activity, with only 32.4% of participants identifying as sexually active. Additionally, 21.91% chose not being sexually active as their reason for not receiving the vaccine, while 16.67% believed they were not at risk of exposure to the virus, and 16.05% felt the vaccine was unnecessary. This further suggests misunderstanding of the importance of vaccination prior to sexual activity, additionally stressing the need for education of this population.

Additionally, it is worth noting that only one‐third of students (33.6%) in our study expressed willingness to take the vaccine in the next year. This figure is relatively lower when compared to the Saudi study, which found that 48.9% of their respondents indicated readiness to take the vaccine [31]. In counterpart, several American studies revealed that more than half of male college students had no intention of getting the HPV vaccine [32]. The latter study also mentioned that no consistent variable was associated with vaccine hesitancy among 39 American studies about this topic [32]. These low numbers of readiness to take the vaccine can also be attributed to the lack of knowledge concerning the potentially harmful consequences of the virus and uncertainties regarding the vaccine’s effectiveness and safety, as hesitancy rates are as high as 76% among college males due to common beliefs that they do not need the vaccine [42, 43]. In terms of social characteristics, our study found an association between higher maternal education levels and increased rates of vaccination. Remarkably, 15 out of the 17 vaccinated subjects in our study had a maternal university education level or higher. This contrasts with a nonsignificant difference concerning paternal educational levels, and as mentioned above, featuring the role that usually mothers play as decision‐makers regarding their children’s vaccination and overall health in general. The latter association is logical and was also noted in Morocco where vaccine acceptability was higher among mothers with higher education levels [44]. These findings emphasize on the importance of focusing on mothers, as maternal education has been identified as the strongest predictor of HPV vaccination uptake among adolescents [45].

On the scale of sexual behavior, we found that HPV vaccine uptake was higher among MSM and students engaging with two or more partners. These two groups fall under the category of high‐risk population, who tend to have higher comprehensive knowledge and acceptability about HPV and its vaccine. While their knowledge scores were slightly higher than those of other students, the difference was not statistically significant. This suggests that factors beyond knowledge alone may influence vaccine uptake in these groups, such as perceived risk or targeted physician recommendation.

Finally, we mentioned in our results that vaccination rates were higher among nonalcohol consumers with a prevalence of 11.7%. In contrast, only 4.1% and 2.4% of frequent and occasional alcohol consumers, respectively, received the vaccine. Two studies in the USA among adults aged between 18 and 26 years showed variable results. One study also indicated that 26.7% of male alcohol consumers received at least one dose of the vaccine compared to 36.2% among their nonconsuming peers [46]. However, the other study found a notable positive association between the acceptance of HPV vaccination and a past occurrence of excessive drinking [47]. In contrast, an Indian study found that students who reported alcohol use were more likely to receive the vaccine (OR: 1.27, CI: 1.14–1.42) [48]. In this context, while alcohol consumption is often linked with increased risky sexual behavior, the diverse associations with HPV vaccine uptake make the nature of this association not fully understood in the literature and warrant further investigation.

The data collection in our study through a single online survey facilitates the collection of sizable datasets in a both cost‐effective and time‐effective manner, with a high yield. In contrast, we faced limitations in our study; in the first place, we were limited by our small sample size with a low response rate despite the high audience reach, which may limit our ability to draw definitive conclusions from our study; second, the questionnaire contained minimal open‐ended questions, restricting the depth of our insight through only prewritten questions. Additionally, online questionnaires lack the benefit provided by face‐to‐face surveys, limiting the clarification of intricate questions. Moreover, the questionnaire targeted a sensitive subject leading to response bias or survey discontinuation. Furthermore, our team was unable to estimate the response rate due to uncertainties about the link’s outreach. Finally, we used the “snowball” sampling technique leading to potential selection bias.

## 5. Conclusion

In conclusion, the impact of HPV‐related cancers in men represents a significant yet frequently neglected public health concern globally and especially in Lebanese society; this is represented by the lack of papers tackling Lebanese men’s health regarding HPV. We conducted a study on 324 male university students in Lebanon aged from 17 through 30 years as this age range falls into the vaccine target population. Our study found a concerning low vaccination prevalence of 5.2%. Suboptimal levels of HPV virus and its vaccine knowledge scores and insufficient physician recommendations highlight the urgent need for targeted HPV vaccine awareness campaigns and educational initiatives in Lebanon for young adolescents, their parents, and healthcare providers, from different public and private sectors. Further research is essential to establish robust evidence supporting the inclusion of HPV vaccination in the national vaccination card, which would standardize physician recommendations and facilitate insurance coverage of its cost, enhancing accessibility and affordability. Addressing these challenges to improve vaccination rates, reduce HPV‐related cancer risks, and advance public health in Lebanon.

**Nomenclature**
ACIP: Advisory Committee on Immunization Practices
CDC: Centers for Disease Control and Prevention
NDS‐UH: Notre Dame des Secours University Hospital
CI: Confidence interval
FDA: Food and Drug Administration
HPV: Human papillomavirus
MSM: Men who have sex with men
NIH: National Cancer Institute
ORa: Adjusted odds ratio
SD: Standard deviation
STI: Sexually transmitted infection
URL: Uniform resource locators
USA: United States of America
WHO: World Health Organization

## Ethics Statement

The study was reviewed and approved by the Institutional Review Board of Notre Dame des Secours‐University Hospital (NDS‐UH IRB) on December 13, 2022.

## Disclosure

All authors have read and approved the final version of the manuscript.

## Conflicts of Interest

The authors declare no conflicts of interest.

## Author Contributions

Ziad Koberssy was responsible for writing the manuscript; Ziad Koberssy, Joviane Daher, Joey El Khoury, and Rami Halabi were involved in sampling and data collection; Rami Halabi and Joey El Khoury conceptualized the study and contributed to the literature review; Charbel El Hachem, Raghid El Khoury, and Nadine Saleh contributed by monitoring the data collection and reviewing the manuscript. Joey El Khoury had full access to all of the data in this study and takes complete responsibility for the integrity of the data and the accuracy of the data analysis.

## Funding

No funding was needed for this study.

## Data Availability

The datasets used and/or analyzed during the current study are available from the corresponding author on reasonable request.
